# The Germanic Model of Liability for Diseases of Animals in Sale Transactions: Historical Heritage or the Dead Weight of Past Generations? Factors Affecting the Form of Legal Standards for Warranty

**DOI:** 10.3390/ani14111669

**Published:** 2024-06-03

**Authors:** Andrzej Dzikowski

**Affiliations:** Department of Food Safety and Public Health Protection, Institute of Veterinary Medicine, Warsaw University of Life Sciences, 02-787 Warsaw, Poland; andrzej_dzikowski@sggw.edu.pl

**Keywords:** civil law, comparative law, warranty, veterinary law

## Abstract

**Simple Summary:**

Latent physical defects in animals sold are problematic for both parties to the contract. This situation implies a legal reaction and increases the seller’s civil liability. One of the types of such liability is the Germanic model of warranty, which is, or was, in force throughout Europe. The characteristics of this model and the conditions which shaped the current statutory acts are demonstrated based on contemporary (Austria, Belgium, France, Luxembourg and Switzerland) and historical (Germany and Poland) examples. The analysis shows factors influencing these legal rules. Local habits of animal trade and law are shown to be decisive factors for the Germanic model.

**Abstract:**

The subject of the analysis is the Germanic model of liability for the physical defects of animals examined through examples in Europe. Methods of legal analysis and interpretation are used. Contemporary (Austria, Belgium, France, Luxembourg and Switzerland) and historical examples (Germany and Poland) are examined and described. The characteristics of this model and the historical conditions which shaped the current legal state are demonstrated. It is shown where particular civil law systems in Europe have maintained the Germanic model of warranty to this day, where other systems have replaced it with another model and what factors have influenced this. The analysis is comparative in regard to legal systems and oriented toward veterinary science.

## 1. Introduction

The problem of the occurrence of diseases as latent defects in sold animals is timeless and universal. For centuries, it has implied a legal response. Three main types of legal response are distinguished: the warranty system derived from Roman law [1,2,3], the Anglo-Saxon model of a breach of obligation without its classification [3,4,5], and the Germanic model of liability for the physical defects of animals.

The source of warranty law is closely related to animals and their state of health. While in the case of Roman law, the standards were generalised and applicable to all sale items, resulting in general institutions of warranties and guarantees, in the case of the Germanic model, the close relationship between the legal standards and the state of animal health was maintained.

In contrast, the model of a uniform concept of a breach of an obligation is not related to the state of health of the animal being sold but only to the upholding and completion (the due fulfilment) by both parties of all elements of the contract entered into.

All three models are currently used in different countries around the world. Apart from “pure” models, there are also mixed systems that integrate different selected elements of the three basic regimes in different ranges of cases [3,6].

This article aims to examine the civil laws of animal warranty of Germanic origin in Europe. The analysis will serve to demonstrate the characteristics of the Germanic model. The historical conditions which shaped the model, its sources and its genesis will be indicated, taking into account the factors that exert influence on the contemporary mechanisms of liability for defects and the activities of official veterinary position holders in Europe.

The subject of the analysis comprises examples of the Germanic model of liability for physical defects of animals, countries where this model is currently in force and other countries where it was still in effect until relatively recently but was replaced by different regulations [7,8,9,10,11,12,13,14,15,16]. Thus, the analysis covers Austria, Belgium, France, Germany, Luxembourg, Poland and Switzerland.

The aim of the research is to answer the following questions: Why did this happen? Why has this model been maintained, or why has it been replaced? What factors influence the maintenance or abandonment of the Germanic model in any particular national civil law system in Europe?

## 2. Materials and Methods

The research material consists of the legal acts of selected countries where the application of the Germanic model of warranty has been shown: Austria, Belgium, France, Luxembourg and Switzerland (where the relevant acts are still current), and Germany and Poland (where the regulations according to the Germanic model were repealed relatively recently) [17,18,19,20,21,22,23,24,25,26,27,28,29,30,31,32,33,34,35]. The territorial scope of the research is limited to European countries, and the temporal scope to the 20th and 21st centuries.

The research is interdisciplinary, comparative in regard to legal systems and oriented toward veterinary science. Juridical scientific and juridical historical methods (using linguistic, functional, teleological, systemic and comparative inquiry) and veterinary medical historical methods are used.

## 3. Results

### 3.1. General Features of the Germanic Model

For the comprehensibility of the results, it is necessary to provide preliminary terminological comments and define the Germanic model.

In the German language literature, it is called the “German model”, “warranty according to German law” and “German principium” (Ger. *deutsch-rechtliches Prinzip*). Naming the model in this way can be claimed to be inappropriate because it suggests that it is characteristic only of Germany or countries where German is spoken. However, the history, scope and genesis of the topic under consideration indicate that the correct name is the “Germanic model”, since its rules derive from the customary laws of the Germanic peoples of the early Middle Ages. This does not mean, however, that the rules of this model were applied only in countries inhabited by ethnically Germanic peoples. The model was also used in Slavic and Romance-speaking countries and in many mixed regulations.

The analysis carried out served to identify the characteristics that can be considered the definition of the Germanic model. In different countries, laws and times, these elements constituted varying proportions of the warranty system and varied in their strength. However, all of them can be considered typical in the light of the results obtained in the current analysis.

The first basic feature is the guiding principle of the assumption of risk by the buyer. The principle is defined as caveat emptor but expressed most often in proverbial form, e.g., *Augen auf*, *Kauf ist Kauf*, or *Bei Pferdehandel und Rinderkauf*, *tut Augen oder Beutel auf* [3,36,37,38,39]. Similarly, in Anglo-Saxon countries, the principle might be expressed as *the buyer’s eye is his merchant* [3,4,5]. This means that the buyer of the animal assumes the entire risk of it possibly having defects. He should examine the animal before entering into a contract and reach certainty that it is healthy and that he wants to buy it for the given price.

Another feature is the existence of specific rules for only a few animal species, primarily horses. Other animals and any other things sold are subject to different rules—termed “general principles”—mostly based on Roman law. This creates a duality of legal liability regimes within a single state. Sales of selected livestock species are subject to specific regulations in accordance with the Germanic model, while most sale contracts are governed by co-existing different general standards. An additional cause of the assorted nature of the sources of law in a single legal regime is the typical dispersion of specific provisions over separate legal or regulatory acts [39]. The species catalogue is accompanied by the distinction of a narrow, casuistic, enumerative catalogue of species-specific major defects, i.e., diseases or stereotypies.

In the Germanic model, there are often locally specific remedies available to the buyer. In addition to two typical remedies—redhibition and price reduction, which originated in ancient Rome—in the Germanic model, there is, e.g., the exchange of an animal for another free of defects or restoration to soundness of the sale object. It is common to limit the availability of traditional entitlements to exclude the possibility of making a price reduction.

The last characteristic which the findings distinguish is the shortness of time limits for the appearance of the disease and for the buyer to use the warranty.

### 3.2. Regulations in Selected European Countries

It was found that the Germanic model still shapes the current Austrian and Swiss regulations controlling warranty in cases of animal defects [21,23,24,26,32,33]. The second tight and distinct group of Germanic model adherents is France, Belgium and Luxembourg [17,18,20,22,25]. The current German warranty law [19,35] is, however, no longer classifiable with the laws of this group, although previously Germany was the most typical example of this solution [34]. Like German law, Polish law has now rejected this model after a long period of Germanic model regulation [27,28,29,30].

#### 3.2.1. Austria

With regard to warranty, it is often considered that the norms of the Austrian civil code [23,40,41] remain in the Roman tradition [42]. It only appears so on the surface. The Austrian system contains many unique elements as well as components of the Germanic principium. It can be characterised as a mixed Romano-Germanic warranty regime [8,43]. This applies to the general rules as well as to the special regulations concerning the defects of animals, which, however, are more Germanic [8,23,32,33,37,43,44,45,46]. The liability for latent defects in animals has been amended several times, most recently in 1972 [32,42,46,47].

Initially, a defect was presumed if the animal died within 24 h of delivery. Currently, a defect may be presumed to exist if it was revealed within six months, and this concerns any defect [23,40,41,46,47]. The possibility of exploiting such a presumption, and therefore of a reversal of the burden of proof, was conditional on the completion by the buyer of due diligence: reporting the detection of a defect, making the animal available for examination by veterinary experts and lodging a security [23,44].

In Austrian law, a certain sequence of rights for the buyer is accepted [23,37]. In the first instance, a buyer can only exercise the entitlement to repair or replacement in circumstances where there is a general, fundamental, complete and permanent impossibility of undertaking the commitment to make repairs (understood as medical treatment of the animal).

When the implementation of the initial remedies is impossible or disproportionately cost- and effort-intensive [23,48], or when the seller refuses to effect these remedies under the buyer’s entitlements or fails to deliver them within the specified period, the buyer can take advantage of a price reduction, and when the defect is not insignificant (*sic*), they may withdraw from the contract.

The time limits for the exercise of entitlements for animals covered by specific provisions are short periods [32,33] (Table 1), and for other species, they are six months from the date of the animal’s delivery [8,23,24]. When animals, as consumer goods, are sold, the period for the presumption of a defect is six months, and the limitation period for warranty claims is two years [8].

Originally, the catalogue of major defects was defined in the code, but now it is defined by the 1972 decree [8,23,32,39]. The erstwhile catalogue’s and present decree’s contents regarding defects are casuistic, encompassing a total of 26 disease and para-disease units (stereotypies) of horses, donkeys, mules, hinnies, cattle, sheep, pigs, rabbits and poultry, and they allot periods for the appearance of the defect from 7 to 150 days. The addressing of infectious diseases in birds should be noted—it is unprecedented in other Germanic systems besides the Austrian system but extremely important in modern large-scale breeding. The Austrian catalogue of major defects is shown in Table 1.

The norm applying only to animal sales is the possibility to sell defective specimens and deposit the amount with a court. This is a court decision at the request of one of the parties if the animal has been examined previously [23].

The warranty system in Austria follows the rules laid down in this code at the beginning of the 19th century [43] and revised at the beginning of the 20th century. It should be considered today somewhat anachronistic. In particular, the Austrian legislature, unlike the German one, sought to preserve the former national law to the maximum extent and only to comply with the requirements of European Union consumer directives [49,50] to the minimum extent [8]. These directives introduce an obligation for EU member states to comply with the goal set by consumer protection, but each state is free to introduce its own means of achieving this goal. Moreover, countries can adopt solutions that are more beneficial to consumers.

#### 3.2.2. Switzerland

The Swiss standards on animal defects consist of statutory standards [21] and implementing regulations [26]. Therefore, a lex specialis according to the Germanic principium is established. The general provisions [21] apply to almost all sale items, including different animal species—horses, donkeys, mules, cows, sheep, goats and pigs excluded.

That regulation means, in principle, the exemption of Swiss sellers of horses, donkeys, mules, cows, sheep, goats and pigs from bearing any liability [21]. Liability is excluded except in cases of the fraudulent concealment of a defect or when the acceptance of such liability is expressed by the seller in writing [21]. A written guarantee may relate, in particular, to the pregnancy of a female animal and the parturition date.

In the Swiss system, unlike other acts in the Germanic model, there is no catalogue of major defects. No disease entities representing latent defects or any specific periods have been enumerated. The implementing act, however, lays down a warranty procedure, which includes notification to the cantonal administrative authorities of the appearance of the defect, the appointment of an expert veterinarian on the official list and the commission of an official veterinary examination of the animal [21,26,39,51].

Swiss law in its current form derives from earlier national and cantonal regulations of the 19th century and retains their basic characteristics, including the procedure for state veterinary authority and expert veterinarian intervention [52].

#### 3.2.3. France

In France, there are general standards of warranty in the civil code [20] based on the Roman model. In addition, there are specific regulations concerning major animal defects in the Rural Code and its implementing provisions, which have been amended several times [22,25,53]. These circumstances make French warranty law heterogeneous and complex. In practice, there are many contradictions between the general and specific rules, and there is often a cumulation of claims under different rubrics. The 2021 amendment was an attempt to unify the legislation regulating the sale of consumer goods and animals [25,53,54]. The amendment did not, however, succeed in unifying the French legislation, which still is a complicated, polyetiological system.

The French catalogue of major defects is extensive and includes many species [53]. It applies not only to the stock farm animal types but also to carnivorous household animals, i.e., dogs and cats. This stands as an important innovation of modern French law. The regulation of warranty in the framework of the Germanic and Roman models concerned primarily beasts of burden. They did not include carnivorous animals at all. In comparison with the unique feature of Austrian warranty law referred to earlier (its extension to defects of poultry), this unique feature of French warranty law is different in a significant way: these carnivorous animals are not kept primarily for economic purposes but are companion animals. The catalogue from the French legislation is shown in Table 2.

There are dedicated, official, accredited veterinary procedures for the diagnosis of infectious diseases, laboratory tests, and clinical examination. Examination by a veterinarian is highly important for warranty in France. It is a mandatory prerequisite for the animal warranty procedure. It is the basis for issuing an expert opinion and deciding on the defect, its occurrence and timing, the amount of price reduction and the reduction in the value of the animal. In addition to official methods, many scientific and practical principles have been laid down in French warranty law that integrate law and the knowledge and art of veterinary physicians.

If the parties are unable to resolve the dispute themselves based on the freedom of civil relations, a lawsuit is necessary. The participation of expert veterinarians is mandatory, as it is in Switzerland [52,53,55]. The buyer of the animal must initiate the procedure for the appointment of medical experts on pain of the action becoming inadmissible. The court immediately appoints one or three expert veterinarians [22,53]. The experts are obliged to take action thereupon, which comprises examining the animal, collecting all pertinent information, recording their judgement and opinion and swearing an oath [22,53]. An examination is not necessary only in the event of withdrawal from the contract for the sale of slaughter cattle due to the livestock’s infection with bovine tuberculosis [22]. In addition to these modes of involving expert veterinarians, since the 19th century, it has been possible to appoint them as private judges [53,55]. A private judge is an arbitrator appointed by the parties themselves who agree to submit to such a jurisdiction; it is a type of alternative dispute resolution. A judgment issued in such informal proceedings is binding and must be enforced in the same way as a judgment of a state court of justice. The appointment of a veterinarian as an arbitrator means that the medical veterinary opinion is also a binding judgment. This is a faster and cheaper way to settle an animal warranty dispute.

These are all characteristic examples of theoretically based but practically applied forensic veterinary medicine. Both the participation of a veterinary expert in the process and, above all, the veterinarian adjudicating the warranty case fulfil the basic features of forensic veterinary medicine: the use of veterinary knowledge in a legal context. At the same time, this specialised knowledge directly influences the shape of the final judgment.

In analysing French warranty law, it is necessary to refer back to its sources. This allows an understanding of why the law in that country is in its present state. Previous findings [55] reveal that Germanic animal laws were a common phenomenon throughout France and were typical of French legal history. Historical–legal analysis of local, pre-revolutionary laws in France provides evidence for the continuity of Germanist regulations [55]. They replaced or supplemented earlier Roman legal norms that were in force even after the fall of the Western Roman Empire. They all derive from the customary laws of the Germanic peoples. It can therefore legitimately be argued that the reason for the current state of the law is the Germanic origin of the Franks, the tribe to which the ancestors of the French of the present day belonged.

#### 3.2.4. Belgium

The major defects of eligible livestock are defined in the 1987 regulation, as amended several times, most recently in 2019 and 2024 [18,53]. The catalogue covers horses, donkeys, hinnies, mules, cattle and sheep and includes contagious diseases and infections with infectious agents, as shown in Table 3.

It was found that it is important to distinguish between “infection” with a given virus and “disease” (as in French law). This is a significant fact from a veterinary and legal point of view and, theoretically and equally, a practically important matter for buyers of animals. Time limits are calculated from the moment the animal is delivered.

It is a point in its favour that Belgian legislation contains provisions that specify the principles of a precise methodology for the official confirmation of infectious diseases (e.g., bovine tuberculosis, brucellosis, enzootic bovine leukosis, herpesvirus infection, equine infectious anaemia, paratuberculosis and bovine viral diarrhoea), as well as the criteria for official laboratory approval.

Termination of the contract and return of the animal are not allowed when the price of the animal exceeds 250 EUR [18]. This provision may be regarded unfavourably because of the pecuniary restriction in the condition. In addition, redhibition is precluded for outbreaks of herpesvirus on cattle fattening and calf farms [18]. This provision is also unfavourable because it differentiates buyers’ positions in an unfair and casuistic manner.

An analysis of Belgian law reveals its historical dependence on French law [53]. This relates to the adoption of the French civil code (the Roman model) as its general principles of warranty and the subsequent (mid-19th century) introduction of specific legal acts (the Germanic model) [53]. While the first instance of taking up an existing legal system can be equated with actions by many other countries that adopted the Napoleonic Code for political reasons, the second can be regarded as legal dependence on an economically stronger neighbour, as was the case with the modelling of Polish regulations on German law. The current Belgian legislation does not rely on French law for its details, and regulations currently in force in both countries differ, but the general idea of the Germanic *lex specialis* remains unchanged.

#### 3.2.5. Luxembourg

Analogous conclusions as for Belgium can be reached by studying the laws in Luxembourg. They are not as modern as in France or Belgium because they come from a 1936 decree, which is, in fact, a revised version of the earlier regulations [17,53]. The catalogue of defects applies to perissodactyls, cattle, sheep and pigs and is shown in Table 4.

There is a presumption of the existence of a defect at the time of the conclusion of the contract for the sale of the animal. The burden of proof falls oppositely from the normal pattern. This means that in the case of enumerated major defects, it is not the buyer who has to prove that the animal had a latent physical defect at the time of sale. On the contrary, it is the seller who must prove that the animal was healthy and sound at that relevant moment. Such a regulation favours the animal’s buyer, which differs from the typical features of the Germanic model. It is important to note that the seller has the possibility of absolving himself of liability if there has been contact between the animals sold and individuals with an infectious disease.

Luxembourg legislation also applies to animals for slaughter [17]. If the defects have rendered the animal unfit for its intended use, i.e., the meat was unfit for consumption, then the buyer can terminate the contract. In cases of conditional or partial fitness for consumption, a price reduction is allowable.

As in France, the legislation in Luxembourg also provides for the appointment of three veterinary experts [17]. Warranty time limits are also the terms within which expert veterinarians are to be appointed (see Table 4), on pain of loss of privileges. This loss may also result from failure to take action within 24 h of the death of the animal as a result of the defect.

#### 3.2.6. Germany

Like the French, the German civil code initially provided general warranty provisions in accordance with the Roman model [19,55,56]. In addition, there were specific provisions in relation to animal defects—based on the Germanic rule [19,34,38,55,56,57]. For a catalogue of the major defects that were recognised in Germany, see Table 5.

It was found that the decision to subordinate certain animal species to the Germanic model was wrong. This is a universal and unanimous opinion in academia [8,13,36,37,38,39,55,56,57,58,59].

What were the reasons for the decision by the German parliament taken at the end of the 19th century? In addition to the influence of pressure groups, especially large-volume traders, the habituation to this form of legislation in most German states was decisive. Such laws had been in force for hundreds of years in most of the states of post-reunification Germany [8,36,43,56,57]. It was not considerations of the rationality of the legislator nor axiological, dogmatic or scientific reasons that swayed the decision.

The contents of the catalogue of major defects in Germany [34] were erroneous, unscientific and anachronistic even at the time of its creation. However, they were not changed for more than a hundred years, until 2001 [35]. Besides the lobbying by breeders, the mythologisation of the civil code can be pointed out among the reasons for the legislation not having been revamped [36,37,38,39,55,57]. The concept of the mythologisation of the civil code is present both in social and legal discourse. According to it, the civil code is a special and unique legal act, more important than other statutory acts. Additionally, it is wrongly believed that it is more difficult to change than other laws or that such a change would have drastic consequences. The result is actual amendment obstruction, such as was recorded in Germany.

The desynchronisation of the legal norms with the exigencies of life grew over time, with the development of veterinary medicine and with changes in animal utility [8,36,60]. In practice, animal buyers and sellers tried not to apply the legal norms at all. Instead, veterinary examinations of animals were carried out, and opinions were drawn up [8].

So why was the change to the regulations finally achieved? The final impetus was the need to adapt to the requirements of European consumer law [7,8,9,10,11,12,13,14], dealing with responsibility in various consumer legal relations, including the consumer sale of goods. The narrow matter of animal warranty reform was far from all that this need concerned. A comprehensive reform of all civil law was carried out. Among a number of changes, specific regulations relating to the physical defects of animals were repealed [7,8,11,13,39,51,58,60]. Thus did the rules of the Germanic model cease to apply after over a thousand years in Germany [9,35].

At the same time, the uniform concept of breach of an obligation and the norms of European consumer law [49,50] were taken up. While consumer standards are intended to be consumer-friendly and to protect the consumer as the weaker party in a commercial relationship, they could also be accepted in the old version of the German civil code. However, the same amendment adopted the Anglo-Saxon idea of a breach of obligation, introducing significant innovations from the common law into the continental law. When modernising the law of contractual obligations, neither the specifics of animals as living organisms nor the advancement of modern veterinary medicine were taken into account at all [39], with the entire system of regulation being adapted to that in place for products of industry.

#### 3.2.7. Poland

As independent Polish laws were being drafted in the interwar period of the 20th century, it was decided to create a comparative civil code. The general principles of warranty were mainly based on French and Swiss law, with Austrian elements [23,29,43], while the animal-specific provisions [27] were a simplified form of German law from the end of the 19th century [34]. Due to this, they were anachronic and unscientific. An additional problem was the imprecise wording of the animal-specific provisions [27] caused by their poor translation from German.

The range of sale material under specific regulation was narrowed down a great deal in Poland in 1966, from which time onwards the rules concerned only horses, mink and sheep [28,30]. Poland was the only country where disease in mink (as fur animals) was particularised.

The original catalogue of defects [27] is shown in Table 5, and the revised 1966 version [28] is shown in Table 6. It was found that neither the terminology nor the definitions were representative of veterinary scientific knowledge. These defects were uncommon and not significant in practice, and some were actually eliminated as a result of the advancement of veterinary science.

Additional, casuistic acts of due diligence were established [28], the non-commission of which resulted in the loss of entitlements (see Table 6). There is no reasonable justification for these requirements.

The almost 50-year endurance of these provisions in the Polish legal system is a counterexample to the rationality of the legislator and the extent to which the laws in effect were suited to scientific knowledge, the needs of civilian life and economic activity.

The novelisation of the legislation in 2014 [30,31] was introduced coinciding with its adaptation to EU legal requirements [49,50]. The overriding aim and cause were the streamlining and internal integration of legislation [61]. Elements of a uniform concept of a breach of an obligation were introduced. It was an imitation of the German amendment of 2001, but to a much lesser extent. The amendment was not preceded by any contemplation of the raison d’être of separate animal legislation or by any discussion in the veterinary literature. The conducted research bore out the conclusion that the latest changes in Polish law (as well as those before World War II) were only an echo and effect of transformations in German law and new European trends [62].

### 3.3. Genesis

Regulations on the Germanic scheme apply in some European countries and have been rejected in others. Such rules were typical of the legislation of 19th-century and 20th-century Europe. The conducted research indicated what the sources of the Germanic model of regulation were. It is derived from the customary laws of the Germanic tribes. It became the basis of numerous laws in the Reich and abroad, including in Poland and France.

The oldest example of the Germanic model is the Law of Bavarians drafted in the middle of the 8th century A.D. [36,39,57,63,64]. The oldest surviving catalogue of major defects in horses and other “cattle” included moon blindness (equine recurrent uveitis), hernias, epilepsy and “leprosy” (lepra equorum—glanders). For warranty action, the major defects should have been latent at the time of sale and revealed themselves within three days and three nights of entering into the contract [63].

The catalogue of major defects may well be considered to be an extremely conservative legal institution. The diseases mentioned in it, such as glanders and equine recurrent uveitis, remained major defects until the 20th century. The terminology and definitions of the catalogues remained unchanged for centuries and completely unrelated to the development of veterinary knowledge. These are examples of anachronism and obscurantism, which worsened over time, reaching their culmination at the end of the 20th century in Poland and Germany [62,65,66].

This model was found to be based on axiological and legal premises which were different from those of the Roman model. The ratio legis was a desire to quickly obtain legal certainty and minimise the possibility of claims.

## 4. Conclusions

This comparative analysis has shown that the regulations of the Germanic model are currently in force in very few European countries. However, these are economically important countries with an advanced legal culture. The existence of these regulations is conditioned on tradition and local customs. Attempts were made to harmonise or approximate the legal rules on warranty, particularly across the common market of the member states of the European Union [65].

It was found that the main progenitor of the analysed model is local legal practice and habit, regardless of whether it goes back 1000 or 150 years. At the same time, deficiencies are sometimes imputed to this model in countries where it has been in force since its inception, as in Germany. Therefore, the endurance of the Germanic model is not shown to be dependent on the length of its duration in force in a particular place. Also, the impact of European law [49,50] is not a sine qua non factor for change, even if it is a direct driver.

It was also found that many different factors cumulate in the rejection or maintenance of the model. In light of the results obtained, it can be concluded that the decisive factor for the maintenance of the Germanic model is local custom (but not what is “customary law” in the legal sense). Among the reasons for the model’s rejection, the desire to modernise and Europeanise the law is at the forefront. Reviewing the results obtained, however, considerations of the model’s equitability, legislative correctness or compatibility with veterinary knowledge of the time are not factors.

The research carried out supports the conclusion that the idiosyncrasies of the Germanic model derive not only from the deficient veterinary knowledge of ancient times but also from the legal emphasis on the completely different interests of the parties to the contract of sale than in the case of Roman law. All legal systems provide conditions for protecting the interests of the parties to the contract, but they value them in different ways and do so with different axiological or praxeological motives. Different solutions are not different as a result of some objective, necessary reasons but rather by virtue of local legal “tradition” or ordinary habit [55,66,67].

While the aim of Roman law and most modern legal systems is to restore the equivalence of benefits disrupted by the occurrence of a latent physical defect in the animal (which is mistakenly identified as favouring the buyer), all the while balancing the legal positions of both parties, the Germanic laws did indeed favour the seller, but not with the seller’s interest as the highest value, but in order to ensure legal certainty (*Rechtssicherheit*) as soon as possible.

The results obtained support the argument that the legal enactments examined allocate value wrongly. It can be argued that they are examples of putting questionable considerations of the rapid achievement of legal certainty and stability in trade above the basic principles of the law of obligations—good faith, equitability, honesty and reliability. It can also be argued that they are characterised by injustice, excessive restriction of the buyer and excessive protection of the seller. These legal enactments may be accused of making unjustified and baseless distinctions between the legal situation of buyers of animals of different species and that of sellers of the same.

This analysis leads to the inevitable question: are the Germanic systems correct in our modern times of advanced veterinary knowledge? Yes, but under certain conditions. Compatibility with current veterinary scientific knowledge is a key condition. Another is to establish effective instruments for defect detection and response. Of course, it is not possible to include all defects in the catalogue. However, if those which are catalogued are verifiably significant medical problems and are committed to record correctly and the prescribed measures are properly implemented, then both sellers and buyers of animals will benefit.

## Figures and Tables

**Table 1:** Major defects of livestock in Austrian law.

Species	Major Defects	Period for Disclosure of Defect
horses, asses, mules and hinnies	recurrent airway obstruction/chronic obstructive pulmonary disease	14 days
	behavioural syndrome correlated with hydrocephalus	
	cribbing and chewing the manger	
	cribbing	7 days
	laryngeal paralysis	
	blindness due to diseases of the inner parts of the eye	
cattle	enzootic bovine leukosis	150 days
	bovine tuberculosis	21 days
	presence of Cysticercus and pulmonary parasites	
	vaginal and uterine prolapse	14 days
	the stereotypy of tongue rolling	
sheep	generalised dropsy with oedematous muscle, with parasite aetiology	21 days
	scabies	7 days
goats	tuberculosis	21 days
pigs	leukaemia	60 days
	cysticercosis	21 days
	trichinosis	7 days
	scabies (including disease caused by *Sarcoptes*)	
rabbits	myxomatosis	10 days
	ear mites	7 days
poultry	Marek’s disease	28 days
	lymphoid leukosis	21 days
	avian encephalomyelitis	10 days
	avian pox	7 days
	Newcastle disease	5 days

**Table 2:** The major defects of livestock and companion animals in French law.

Species	Major Defects	Period for Disclosure of Defect	Time Limit for Bringing an Action and for the Appointment of Veterinary Experts *	Additional Requirements
horses, donkeys, mules	catalepsy		10 days	
	emphysema			
	chronic laryngeal paralysis			
	stereotypy, tics with or without the involvement of teeth			
	chronic lameness			
	isolated equine recurrent uveitis		30 days	
	equine infectious anaemia			established as official procedures stipulate
cattle	bovine tuberculosis in animals which have clinical signs and are reactive in tuberculin test		15 days	
	infectious bovine rhinotracheitis/infectious pustular vulvovaginitis		30 days	
	enzootic bovine leukosis			
	brucellosis			
goats				
sheep			10 days	
pigs	trichinosis		10 days	
cats	feline infectious peritonitis	21 days	30 days	
	FeLV infection	15 days		
	feline panleukopaenia	5 days		
	FIV infection			
dogs	canine distemper	8 days	10 days	
	infectious canine hepatitis (Rubarth’s disease)	6 days	10 days	
	canine parvovirus	5 days	30 days	
	hip dysplasia in dogs less than 1 year of age			confirmed by radiology
	testicular ectopia in dogs over 6 months of age			
	retinal atrophy			

* There are three types of time limits: in addition to the terms for the disclosure of defects, there are two further terms (both of the same length)—for redhibition (for filing an action) and for the appointment of veterinary scientific experts.

**Table 3:** Major defects of livestock in Belgian law.

Species	Major Defects	Period for Disclosure of Defect	Additional Obligations
horses, asses, mules and hinnies	glanders	9 days	
	equine infectious anaemia	30 days	officially confirmed
cattle	rinderpest	9 days	must be the fault of the seller
	preterm calving		
	white heifer disease in breeding animals		
	bovine tuberculosis	15 days	officially confirmed
	brucellosis	30 days	
	infection with BoHV-1		officially confirmed and obtained a non-negative result in a besnoitiosis ELISA test
	enzootic bovine leukaemia		officially confirmed
	paratuberculosis		
	bovine viral diarrhoea (both immunotolerance and persistent infection)		
	neosporosis		
	contagious bovine pleuropneumonia		
	IBR/IPV infection		
sheep	ovine smallpox	9 days	

**Table 4:** The major defects of livestock in Luxembourg law.

Species	Major Defects	Period for Disclosure of Defect	Additional Obligations
parissodactyls	glanders and strangles, understood as one defect	15 days	
	chronic, incurable diseases manifested by an impairment of the animal’s consciousness		
	difficulty breathing due to chronic, incurable lung and heart disease		
	chronic, incurable stenosis of the upper vocal folds		
	cribbing		
	chronic, incurable inflammation of the inner parts of the eye with internal causes	29 days	
animals of the Bovidae family	paratuberculosis	30 days	identified clinically and confirmed by official bacteriological tests
	bovine tuberculosis	15 days	confirmed (cumulatively) clinically and as a result of official tuberculin tests
	haematuria due to damage to the bladder mucosa		
	neosporosis		
	contagious bovine pleuropneumonia		
	infectious bovine rhinotracheitis/infectious pustular vulvovaginitis infection		
sheep	scabies caused by *Psoroptes* spp. and *Sarcoptes* spp. parasites	15 days	
pigs	pneumoenteritis with acute or subacute septicaemia, as well as (possibly) pneumonia, laryngitis, acute colitis and subcutaneous inflammatory oedema, caused by *Bacillus bipolaris suis*	5 days	

**Table 5:** The major defects of livestock in the laws of Germany (until 2001) and Poland (until 1966).

Species	Major Defects	Period for Disclosure of Defect		Additional Obligations
		Animals Not Destined for Immediate Slaughter	Slaughter Animals	
horses (literally, but the interpretation of the term’s purpose here indicates all Equidae)	glanders	14 days */21 days **	14 days *	determined by visual inspection on the basis of external indications or official tests **
	heaves—difficulty breathing due to chronic and incurable lung or heart disease (and therefore more widespread than recurrent airway obstruction/chronic obstructive pulmonary disease)	14 days		
	wheezing (difficulty breathing due to chronic and incurable diseases of the larynx or trachea, manifested by characteristic roaring noises during breathing and of a chronic nature)			
	various cases of stereotypy			
	equine recurrent uveitis—a disease of the inner parts of the eye			
	a behavioural syndrome correlated with a gradual or progressive, incurable brain disease due to hydrocephalus, manifested by impaired consciousness of the animal			
cattle	bovine pulmonary disease	28 days		
	open tuberculosis of the udder **	21 days		
	tuberculosis causing general emaciation of the animal	14 days */21 days **		
	bovine tuberculosis in which more than half of the carcasses were unfit for consumption or were only conditionally fit *		14 days *	
sheep	scabies	14 days		
	generalised dropsy and oedematous muscle due to internal diseases or inadequate nutrition		14 days *	
pigs	trichinosis			
	cysticercosis			
	swine fevers, classical swine fever	10 days		
	tuberculosis resulting in cachexia *		14 days *	
	Erysipelas *	3 days		

* Applies to Germany only. ** Applies to Poland only.

**Table 6:** Major defects of livestock in Polish law (until 2014).

Species	Major Defects	Period for Disclosure of Defect *	Additional Obligations
horses	cribbing	15 days	
	wheezing		official examination in a state medical institution or university veterinary clinic
	a behavioural syndrome correlated with hydrocephalus—a chronic disease of the brain or meninges with impairment of the consciousness of the animal		
	chronic disease of the inner parts of the eye without traumatic cause	30 days	
minks	tuberculosis		
sheep	scabies	15 days	official examination in a state medical institution or university veterinary clinic; the buyer is obligated to notify the state veterinary administration within 24 h

* Within 7 days from the end of the above periods, the buyer was obligated to notify the seller of the defect.

## Data Availability

Data are contained within the article.
